# Cold Sintering Technology as a Friendly and Sustainable Way of Producing Ceramic Materials from Recycled Waste

**DOI:** 10.3390/ma19081512

**Published:** 2026-04-09

**Authors:** Gyorgy Thalmaier, Nicoleta Cobîrzan, Traian Florin Marinca, Mircea Nasui

**Affiliations:** 1 Faculty of Materials and Environmental Engineering, Material Science and Engineering Department, Technical University of Cluj-Napoca, 103 Muncii Blv., 400641 Cluj-Napoca, Romania; gyorgy.thalmaier@sim.utcluj.ro (G.T.); traian.marinca@stm.utcluj.ro (T.F.M.); 2 Institute of Nanomaterials and Nanotechnologies—EUTINN, European University of Technology, European Union, 28 Memorandumului Street, 400114 Cluj-Napoca, Romania; mircea.nasui@chem.utcluj.ro; 3 Faculty of Civil Engineering, Buildings and Management Department, Technical University of Cluj-Napoca, 15 Constantin Daicoviciu Street, 400020 Cluj-Napoca, Romania; 4 Faculty of Materials and Environmental Engineering, Physis and Chemistry Department, Technical University of Cluj-Napoca, 103 Muncii Blv., 400641 Cluj-Napoca, Romania

**Keywords:** secondary raw materials, clay bricks, waste, cold sintering, low carbon

## Abstract

This paper presents a new ceramic building material produced by the cold sintering process (CSP), as a greener and cleaner technology compared to conventional ones. The ceramic composites were made from recycled clay bricks (RCBs), a byproduct resulting from construction and demolition and waterglass (WG) as a liquid solvent, pressed at 400 MPa and sintered at a temperature of 150–200 °C. After the samples were produced, their structure and physical and mechanical properties were investigated. The internal morphology of samples shows a homogeneous structure with a low porosity (up to 3%). The compressive strength of ceramic building material produced by the CSP was up to 211 MPa, considerably higher than the reference sample whose value was 45 Mpa. This high strength is due to the internal structure of the ceramic composed of a tough amorphous phase that acts like a binder holding together the powder particles and closing most of the porosity inside the material.

## 1. Introduction

Ceramic buildings materials have many applications in the construction field, being one of the most used building materials in the world. Resistant to weathering and biological and chemical attacks, ceramic materials have a long service life and low maintenance cost. Produced in a wide range of sizes (tiles, bricks and blocks), ceramic materials require a high firing temperature, to retain their strength, durability and quality. To reduce the footprint of ceramic building materials in the production stage, a series of effective solutions have been applied, such as the replacement of fossil fuel with renewable energy and the integration of circular practices in the production line [1,2,3,4]. The circular economy, considered and adopted in the construction and materials industry, involves a transition towards low-carbon operation, and considers innovative materials and technology as being imperative to overcoming technical, economic and legal barriers [5]. An efficient circular model considers recycling, reusing, or increasing the durability and service life of products, as a responsible way to reduce the landfilling and extraction of natural resources. The potential of using additives (organic and inorganic materials) as fluxing or pore-forming agents to improve thermal properties or to reduce the materials’ footprint was intensively studied in recent years [6,7,8,9,10,11,12]. Additionally, raw materials or byproducts resulting from construction and demolition, or different industries, are generated annually in large quantities and can represent a valuable resource for the construction field if they are recovered and recycled. Bricks, blocks or tiles, as ceramic-based materials, result in a high quantity of waste during their entire life cycle, from the production stage to the construction, rehabilitation or demolition of buildings, requiring landfilling and transport from the site to landfill or a recycle center. These processes are energy consuming, with relevant environmental impacts. After disassembling masonry elements at the end of a building’s lifespan, reclaimed brick treatment (cleaning and sorting) is needed to meet the provisions of standards as a preliminary stage in the reusing process [13,14,15,16,17]. Transforming recycled debris bricks into additives for the production of new building materials [18,19] involves crushing, grinding and sieving to achieve different particle sizes.

The conventional sintering process is dependent on mineral transformations and the diffusion mechanisms of minerals, which take place at different temperatures. The early stage of sintering, usually at a lower temperature than 800 °C and mainly caused by the surface diffusion mechanism [20], is the stage where the pores remain interconected and open and materials have low mechanical strength. At a higher temperature, over 950 °C, the sintering process becomes more complex; the grain boundray and lattice diffusion mechanisms along the clay particles appear, which promotes the material’s densification process [21] and the formation of a glassy phase (vitrification). Pores become isolated, closed and partially filled by glassy liquid mass. The mechanical properties of ceramic materials are therefore influenced by chemical and mineralogical composition, transformations and the formation of new minerals, and by sintering temperature.

The cold sintering process (CSP) is an innovative method for sintering ceramic-based materials at lower temperatures, as an alternative to the traditional sintering process. Developed in 2016 and patented in 2017 by Clive Randall and his research team from Penn State University [22], this sintering process consists of densifying inorganic compounds, such as ceramic powders, at a relative low temperature, usually under 400 °C. A transient solvent (aqueous acid/base solution) is used to facilitate the mass transfer and dissolution–precipitation process during production [23,24,25,26,27]. The application of pressure and heat at a certain temperature ensures that the solvent evaporates and sustains the entire process by rearranging the particles and facilitating the densification of the material. Using a low temperature for sintering is considered a green innovative technology, which can be effective in the ceramic industry to reduce greenhouse gases (GHGs) toward decarbonization goals.

The CSP, due to the lower energy required in the densifying process, is used in the Automotive Industry for the production of battery components for electric vehicles [28,29] and ceramics (piezoelectric [30], alumina [31], and zirconia [32]).

In this context, the main objective of this research is to develop a new ceramic material based on 100% ceramic waste. Clay waste, resulting from construction and demolition, is found globally in high quantity at low cost, and may represent a valuable resource for the production of new ceramic materials. Using brick powder in the production of new ceramics may generate benefits in terms of the environment (reduce landfilling; conserve natural resources, such as clay, sand or water; reduce CO_2_ emissions during production process, etc.) in comparison to conventional ceramic products. As a sintered material, already fired at high temperature, and having undergone a complete mineralogical transformation it is therefore a good candidate for cold sintering technology. The CSP, a sustainable technology for sintering at lower temperatures, was applied for the production of ceramic materials (CS_i). To highlight their potential of being explored for the production of low-carbon-footprint materials, their structure and technical parameters are analyzed and compared with a conventionally produced reference sample.

## 2. Materials and Methods

### 2.1. Sample Preperation

Recycled clay bricks (RCBs), resulting from the structural rehabilitation of masonry buildings, were collected and prepared (dry, crushed and sieved) for use as a secondary raw material in the ceramic composite produced by the CSP. The particle size of the RCBs obtained was less than 0.1 mm.

Gray clay (GC) was used for the production of reference samples. The mineralogical composition of the clay was expressed using X-ray diffraction presented elsewhere [6]. Sodium silicate (Na_2_O·nSiO_2_) solution, known as waterglass [33], as transition solution with the ability to form silica gels, was added to the ceramic composition at a concentration of 33–35 wt.%.

**Figure 1:**
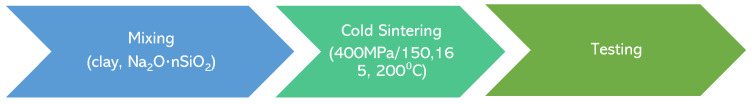
The flowchart of the experimental study.

The recycled clay brick powder was mixed with waterglass (WG) (Figure 1); then, the ceramic materials were cold sintered in a cylindrical die and maintained under temperature (200 °C (CS_1), 165 °C (CS_2) and 150 °C (CS_3)) and pressure (400 MPa) conditions for 30 min.

A reference sample (reference) made of clay, was dry pressed at 39 MPa using a hydraulic press and fired at a temperature of 800 °C/2 h, following the conventional firing technique [34]. The resulting ceramic composites were investigated and tested in the laboratory.

### 2.2. Characterization Methods

Phase identification of the clay bricks after milling was done from the X-ray diffraction patterns achieved using an Equinox D3000 X-ray diffractometer (Co-kα radiation, Inel, Artenay, France). The microstructure of the ceramic-based materials was characterized based on scanning electron microscopy (SEM - Jeol 5600 LV microscope, Tokyo, Japan,) images and energy-dispersive X-ray spectroscopy analysis (Oxford Instruments, Aztec software, version 4.2, High Wycombe, UK). Prior to SEM analysis, the samples were fixed to the sample holder with carbon tape, and a thin gold layer was sputtered onto the surface to increase conductivity. The morphology of minerals and the local chemical composition were also determined by SEM–EDX.

Infrared spectra for the samples were measured on a Bruker Tensor 27 FTIR spectrometer (Karlsruhe, Germany) in Attenuated Total Reflection (ATR) mode.

The density and compressive strength were determined on ceramic samples having a D/H ratio of ~1. The density (ρ) of ceramic materials (CS_1; CS_2; CS_3; and reference) was determined by dividing the sample’s mass (m) by the calculated volume (V). The compressive strength of the ceramic specimens was determined by using a hydraulic press on three samples for each type. The porosity was estimated as follows: $P=1-\frac{\rho_{sample}}{\rho_{material}}$, where the material’s density was measured by water picnometry.

## 3. Results and Discussion

### 3.1. Raw Materials Characterization

**Figure 2:**
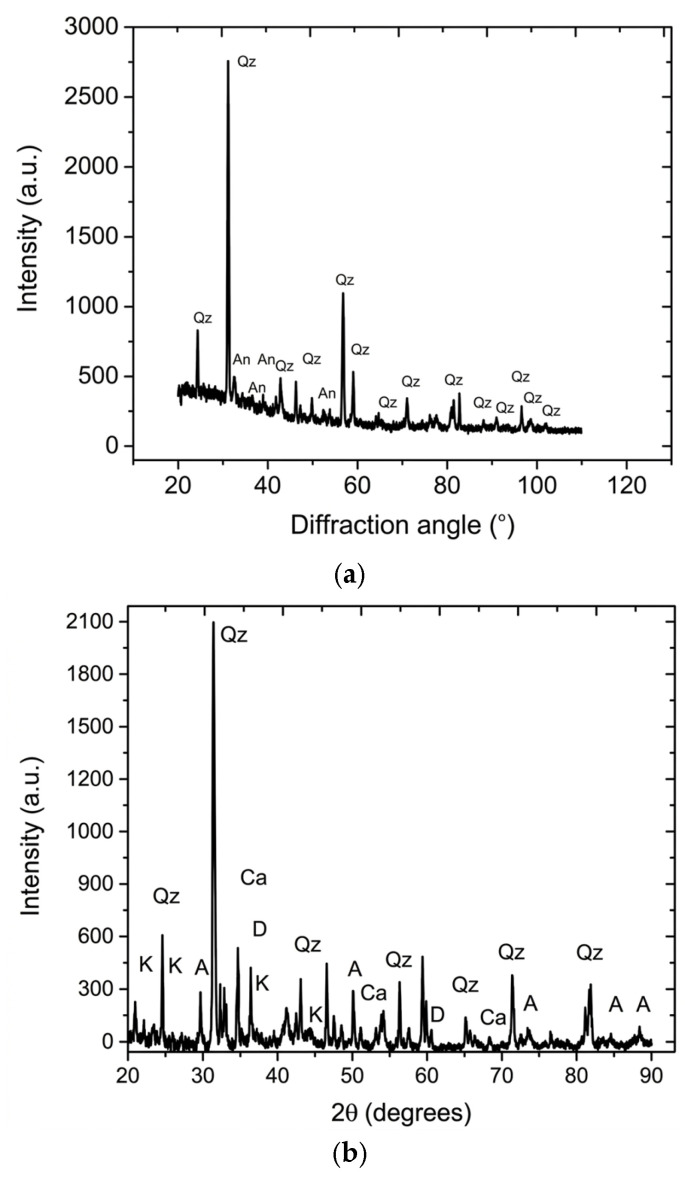
The XRD patterns of starting materials, the RCB powder (**a**) and gray clay (**b**): quartz (Qz), anorthite (An), kaolinite (K), Calcite (Ca), dolomite (D), Atanase (A).

XRD only detects crystalline phases. In recycled bricks (RCBs), a large portion of the material is in an amorphous (glassy) phase formed during the initial firing process, which XRD cannot identify as specific mineral peaks. According to the XRD analysis, the two crystalline phases, quartz (SiO_2_) and anorthite (CaAl_2_ Si_2_ O_8_), are detected in the recycled brick powder, as shown in Figure 2.

The silica content in this material is very high. Most of this remains as “residual quartz”, sand particles that did not react with the other oxides during firing. It is almost always the strongest peak in clay brick XRD. The CaO and alumina in the initial raw material reacted to synthesize anorthite during firing. This usually happens when the calcium from carbonates (like Calcite) reacts with clay minerals (like kaolinite) at temperatures between 900 °C and 1100 °C [35,36]. If minor minerals (like Hematite or mullite) make up less than 2–3% of the total volume, the XRD peaks might be too small to distinguish from the background noise.

**Figure 3:**
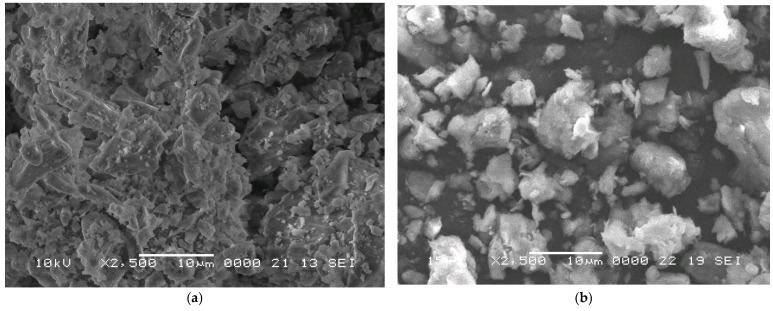
The SEM images of starting materials: (**a**) the RCB powder and (**b**) GC.

The microstructure of raw materials was analyzed using scanning electron microscopy (SEM), as shown in Figure 3.

The chemical composition of the GC and RCB was calculated from the SEM–EDX analysis presented in Table 1. The results show that GC has a high content of SiO_2_ (61.02%), an average content of Al_2_O_3_ (11.82%) and K_2_O (11.87%), and a low content of CaO (6.72%), MgO (4.70%), and Fe_2_O_3_ (3.89%)_._ The RCB has a similar chemical composition to clays. It contains SiO_2_ (62.6%), Al_2_O_3_ (14.6%), Fe_2_O_3_ (11.2%), CaO (5.0%), MgO (1.4%), K_2_O (2.9%), Na_2_O (1.6%), and some traces of TiO_2_ (0.6%). The high content of Fe_2_O_3_ (11.2%) found in RCBs justifies their dark brown color.

The content of SiO_2_ + Al_2_O_3_ + Fe_2_O_3_ is higher than 70% in both raw materials.

The most interesting part in the RCBs is linked with the high content of iron 11.2%, which is not seen as Hematite peaks in the XRD pattern. This phenomenon can be possible due to the following:

- Glass incorporation: Iron acts as a “flux” during the firing process, it melts and remains in an amorphous, glassy state.
- Solid solution: Some iron can substitute for aluminum in the anorthite crystal structure, meaning it is “hidden” inside the anorthite peaks.

Potassium and sodium (summing 4.5 wt.%) also act as fluxes. They lower the mixture’s melting point, promoting the formation of a liquid phase that cools into glass. Since glass has no long-range atomic order, it creates a “broad hump” in the XRD background (visible in the 20–40° area) rather than sharp peaks.

### 3.2. Ceramic Materials Characterization

#### 3.2.1. Structural and Microstructural Characterizations

**Figure 4:**
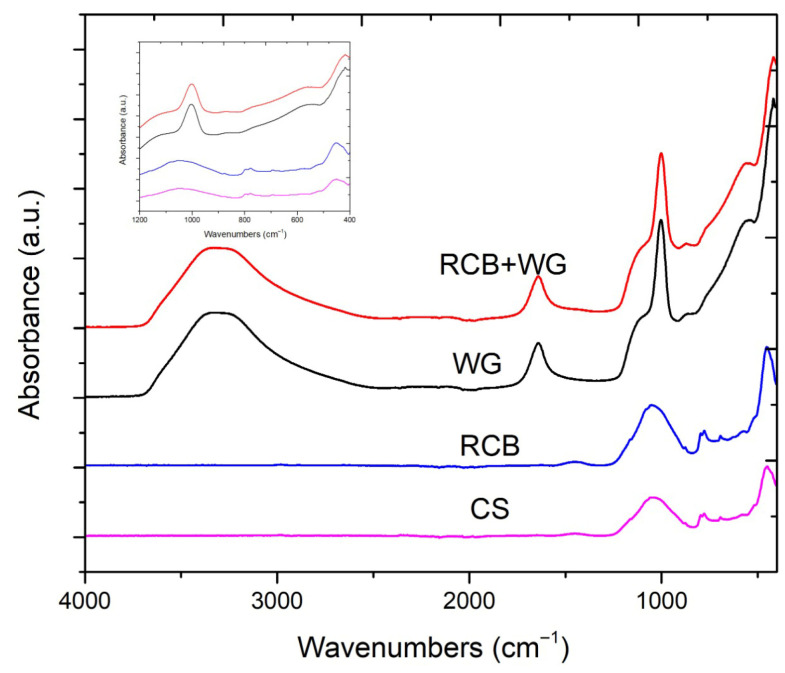
The FTIR spectra of WG (black line), RCBs (blue line), mixture of the RCB and WG (red line), and the CS_2 sample (165 °C) (pink line).

Figure 4 presents the FTIR spectra of waterglass, milled brick, and cold-sintered samples. Clear differences are observed across the spectra, indicating significant chemical and structural changes during the cold sintering process.

The spectrum of waterglass (WG) shows a broad and intense absorption band in the 3300–3400 cm^−1^ region, assigned to O–H stretching vibrations originating from structural hydroxyl groups and adsorbed water present in the sodium silicate solution. In contrast, the cold-sintered sample (CS_2) exhibits an almost flat response in this region, confirming the effective removal of free and weakly bound water. Eliminating residual water is essential for achieving high densification, as trapped water may lead to pore formation during consolidation.

In the 900–1100 cm^−1^ range, characteristic of Si-O-T (T=Si, Al) asymmetric stretching vibrations, the RCBs exhibit a broad band centered around 1050 cm^−1^, typical of quartz and glassy aluminosilicate phases. Waterglass displays a sharp and more intense peak around 1000 cm^−1^, associated with depolymerized and highly reactive silicate species. After cold sintering, the corresponding band decreases in intensity and shifts slightly, indicating the formation of a sodium aluminosilicate (N-A-S-H) network on the particle surfaces. This shift towards lower wavenumbers consists of increased polymerization and the development of a more constrained silicate structure.

The feature previously cited as 1450 cm^−1^ has now been correctly assigned to the band near 1600 cm^−1^, which corresponds to the H-O-H bending mode of molecular water in water glass. Carbonate-related adsorption, typically found near 1450 cm^−1^ in alkali silicate systems exposed to atmospheric CO_2_, are not present in the cold-sintered sample. This absence suggests that sodium ions are incorporated into the aluminosilicate framework rather than forming carbonate phases, indicating that the cold sintering process suppresses carbonation under these conditions.

In the low-frequency region between 450 and 800 cm^−1^, now highlighted in the revised Figure 4, additional differences become evident. These bands are associated with Si-O-Si and Si-O-Al bending models and reflect deeper structural reorganization during the cold sintering and geopolymerization processes. The more defined features of the waterglass and RCB mixture further support the formation of a partially polymerization aluminosilicate network.

**Figure 5:**
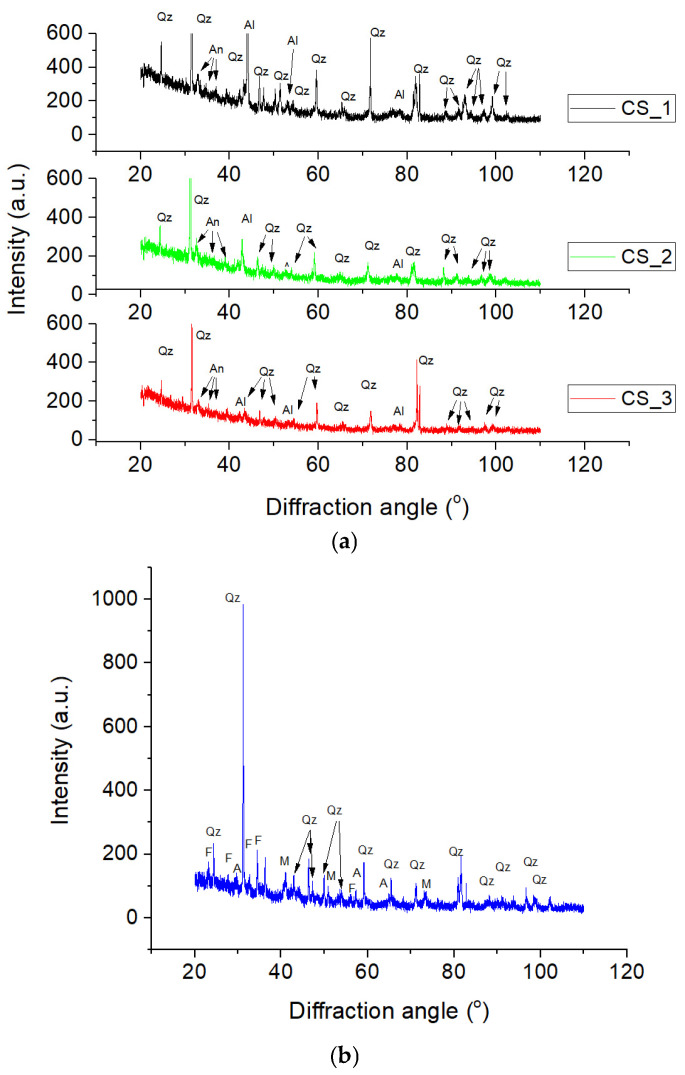
The XRD patterns of (**a**) cold-sintered samples CS_1 (200 °C), CS_2 (165 °C), CS_3 (150 °C), and (**b**) the reference samples: quartz (Qz), anorthite (An), Al-oxide (Al), Feldspar (F), Atanase (A), Magnetite (M). (The XRD patterns in Figure 5a are presented with the quartz (101) peak (~32°) truncated to improve the visibility of the other low-intensity peaks).

The cold sintering process with waterglass as a liquid additive results in a composite pattern that maintains the original brick minerals while introducing new crystalline peaks. The mineralogical composition of the clays expressed using X-ray diffraction by the CSP shows that the samples contain quartz (SiO_2_), anorthite (CaAl_2_ Si_2_ O_8_) and aluminum oxides (Al_2_O_3_), as shown in Figure 5. The most significant change in the pattern is the increasing and sharpening of diffraction peaks (labeled with Al), after the 165 °C and 200 °C treatments, corresponding to aluminum oxide. These peaks correspond to ICDD card no. 80–1385. The ratio of the (101) quartz plane to the aluminum oxide (400) plane was not modified significantly for the cold-sintered samples (~8).

**Figure 6:**
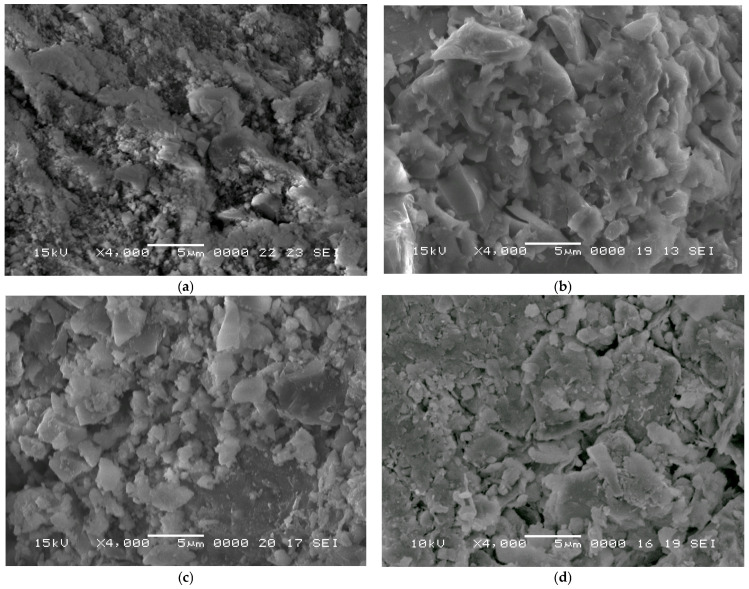
The SEM images of cold-sintered samples (**a**) CS_1 (200 °C), (**b**) CS_2 (165 °C), (**c**) CS_3 (150 °C), and (**d**) the reference sample.

The microstructure of raw materials was analyzed using Scanning Electron Microscopy (SEM), as shown in Figure 6.

The chemical ceramic composited calculated from the SEM–EDX analysis reveals a high content of SiO_2_, an average content of Al_2_O_3_ and a low content of Na_2_O, CaO, MgO, and Fe_2_O_3_ TiO_2_ and K_2_O in all samples, as shown in Table 2.

The transition from the RCB pattern to the cold-sintered pattern illustrates several key chemical effects:

*Alkali Activation:* The waterglass reacts with the aluminosilicate precursors in the brick to form a sodium aluminosilicate (N-A-S-H) geopolymer-like gel. This gel acts as the “glue” or binder that densifies the material at such a low temperature.

*Amorphous Background*: A slight broad “hump” between 20° and 40° is often visible, indicating the presence of this amorphous binder phase or residual sodium silicate glass.

*Preservation of Bulk Properties:* Unlike traditional sintering at >1000 °C, the 200 °C process does not cause the quartz to melt or transform into other mineral phases (like mullite), preserving the recycled brick’s core mineralogy.

The interaction between waterglass and recycled brick particles is fundamentally transformed under the high-pressure conditions of the cold sintering process. In standard geopolymerization (conducted at ambient pressure), waterglass typically results in a slow, surface-level dissolution of the aluminosilicate precursors.

However, at 400 MPa, the mechanical stress is not uniformly distributed; it is highly concentrated at the tiny contact points where individual grains touch. The local stress at these junctions far exceeds the nominal 400 MPa, triggering an almost instantaneous dissolution of the surfaces into the sodium silicate liquid. This high-pressure environment then mechanically forces this “chemically enriched” binder into the microscopic pores of the matrix.

While the dissolved alumina remains as an amorphous gel at room temperature, the thermal energy provided during the sintering stage allows these ions to reorganize. This process facilitates the formation of extremely small alumina nanocrystals, as observed in our XRD patterns. These nanocrystals act as a secondary reinforcement within the amorphous binder phase, effectively “bridging” the larger quartz and anorthite grains and contributing to the material’s monolithic strength.

#### 3.2.2. Physical and Mechanical Properties

Moisture and mechanical stress are responsbile for material degradation. Therefore, ceramic materials must be resistant to environmental factors (salt exposure, freeze–thaw cycles and chemical and biological activities) to meet the essential quality and durability performance requirements during the building’s service life. The porosity of materials plays an important role in controlling moisture and is in a strong correlation with a material’s density.

The transition from recycled brick powder to a dense, high-strength ceramic is primarily driven by the dramatic reduction in porosity achieved through the CSP. As may be observed in Table 3, the porosity of the CSP samples was significantly lower than that of the reference sample (~15%). Specifically, porosity values ranged from 6.6% for CS_3 (150 °C) down to 2.7% for CS_1 (200 °C)—a reduction of up to 82% compared to the reference. These results strongly correlate with SEM observations, which reveal a highly homogeneous internal morphology and a high degree of sintering.

This densification is further reflected in the material’s density, which increased by 11% (2.10 g cm^−3^) and 16% (2.19 g cm^−3^) compared with the reference sample (1.89 g cm^−3^) (Table 3), making this material adequate for application as a ceramic based-material in low-carbon-footprint buildings.

Compressive strength is one of the most important parameters in building material characterization, which, in the case of the conventional sintering process, is influenced by both the sintering temperature and manufacturing process. The compressive strength of ceramic materials usually increases with the firing temperature and when the phase transformation is considered complete [37,38]. Therefore, in the reference samples produced by the conventional sintering process, the resulting density was greater than 1.89 g/cm^3^, which is a normal value for traditional ceramic bricks.

**Figure 7:**
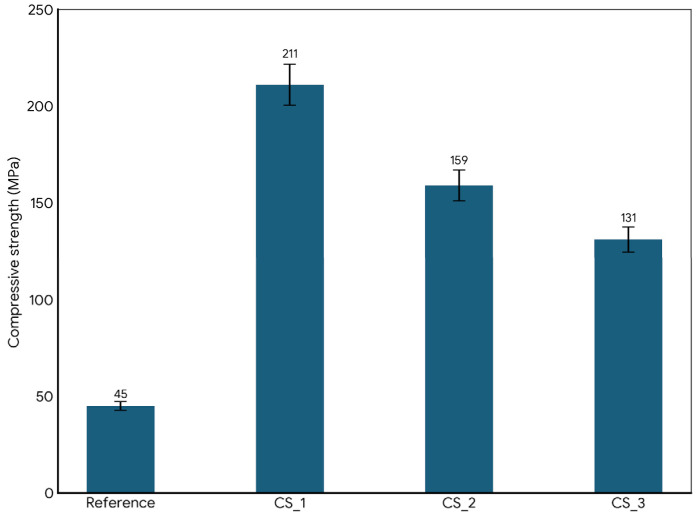
The compressive strength of the reference and cold-sintered samples.

In the case of ceramics produced by cold sintering (Figure 7), an improvement in compressive strength was obtained for all CS samples in comparison with the reference samples produced at a higher temperature (800 °C).

This structural evolution explains the increase in compressive strength up to 370% (reaching 211 MPa for CS_1). The relationship is clear: the 400 MPa pressure and the chemical action of the sodium silicate solvent force the binder into microscopic pores, while the subsequent 200 °C treatment facilitates the formation of reinforcing alumina nanocrystals within the amorphous binder. This optimized structure–property relationship makes these materials exceptionally suited for application as high-performance, low-carbon-footprint building components.

The strength of a ceramic is usually limited by its porosity. Standard bricks have porosity in the 20–30% range so their compression strength is low. The use of cold sintering eliminates most of the porosity by using pressure and filling the remaining gaps with the help of the waterglass brick dissolution/reprecipitation process. After the cold sintering process, CSP samples reach >95% theoretical density. At this density, the material stops behaving like a “brick” and starts behaving like a monolithic ceramic.

According to the Griffith Crack Theory [39], the strength of a ceramic is inversely proportional to the square root of its largest flaw (a pore in our case). In most cold sintering experiments, iron can be a problem because it inhibits certain reactions. However, in this case, the absence of crystalline Hematite peaks in the XRD pattern suggests that the iron is perfectly integrated into the amorphous binder. Iron-rich silicate glasses/gels are known to be tougher and less brittle than pure silicate binders [40]. This tough glass binder is further strengthened by the presence of the small alumina particles and the higher Na content, which helps the samples to reach 200 MPa without becoming too brittle [41,42].

The sustainability of these ceramic-based materials (CS_1, CS_2 and CS_3) is improved in comparison with reference samples, due to the following characteristics:

- *Lower environmental impact* resulting from: the valorization of ceramic waste, the production of new materials, reducing the landfilling and extraction of natural resources (a.1), and using a method which requires a lower sintering temperature (150–200 °C), lower energy consumption and therefore has lower greenhouse gas emissions (a.2) [43].
- Lower energy consumption and therefore lower greenhouse gases emissions (a.2). Considering the measurements made on a laboratory-scale sample and on different laboratory equipment, the energy consumed to heat the sample to the sintering temperature was greatly diminished in the case of cold sintering samples, from about 8 kWh for classical sintering to around 0.4 kWh for the cold sintering process. This reduction arises from the lower required temperature of 150–200 °C (CS_1–CS_3) in comparison to 800 °C (reference). Considering that for each consumed kwh electrical energy, 0.42 kgCO_2_ eq is generated, it results that CS samples resulted in a significant reduction in CO_2_ emissions.
- *Reduces costs due* to C&D recycling, which is an abundant resource (c.1), and reduced firing time (0.5 h) (c.2).
- *Higher mechanical strength* (131–211 MPa) (d.1) and good physical properties (6.6–2.7% porosity and 2.1–2.19 g/cm^3^) (d.2).

## 4. Conclusions

Ceramic waste may be found in a high quantity and represent a sustainable and valuable resource for recycling and waste valorization in the ceramic industry. Therefore, the innovation of this article consists of producing new ceramic building material, based on 100% ceramic waste. The CSP, a sustainable technology for sintering at lower temperatures, was selected for the production the ceramic materials.

The use of ceramic waste and the CSP as green technology are effective approaches in accelerating the transition towards low-carbon practices in ceramic and building industries. The main findings of this experimental study show the following:

- The internal morphology of samples produced by the CSP is a homogeneous structure (see the SEM analysis) with a high sintering degree.
- The compressive strength of samples improved, up to 211 MPa, which represents an increase of about 369% (CS_1) in comparison to the reference sample. Even so, the apparent density increase was low, only 16%.
- A lower carbon footprint of samples produced by the CSP, due to both benefits (recycled waste and reduced heat for the production process, lower than 200 °C).

As may be seen from this experimental study, these new ceramic materials have good mechanical properties with high potential to be used for the production of clay materials (brick, tiles, etc.) for application in buildings.

Further research will be conducted in this field to extend the laboratory research to real environmental conditions.

## Figures and Tables

**Table 1:** The calculated oxide content (wt.%) in the RCB powder and GC according to the SEM-EDX analysis.

Oxides	SiO_2_	Al_2_O_3_	Fe_2_O_3_	CaO	K_2_O	MgO	Na_2_O	TiO_2_
GC	61.02	11.82	3.89	6.72	11.87	4.70	-	-
RCB	62.6	14.6	11.2	5.0	2.9	1.4	1.6	0.6

**Table 2:** The calculated oxide content (wt.%) in the cold-sintered and reference samples according to the SEM-EDX analysis.

Oxides	SiO_2_	Al_2_O_3_	Fe_2_O_3_	CaO	K_2_O	MgO	Na_2_O	TiO_2_
Reference	58.9	16.8	10.1	7.9	1.8	3.1	0.8	0.7
CS_1	70.0	11.8	7.8	3.3	2.2	1.3	3.2	0.5
CS_2	65.1	14.7	7.3	2.9	1.9	2.0	4.3	1.3
CS_3	68.1	11.0	9.0	4.6	2.4	1.2	2.8	0.8

**Table 3:** The porosity and density of the reference and cold-sintered samples.

Properties	Sample			
	Reference	CS_1	CS_2	CS_3
Porosity (%)	15	2.7	3.3	6.6
Density (g/cm^3^)	1.89	2.19	2.18	2.10

## Data Availability

The original contributions presented in this study are included in the article. Further inquiries can be directed to the corresponding author.
